# Trade-off between competition and facilitation defines gap colonization in mountains

**DOI:** 10.1093/aobpla/plv128

**Published:** 2015-11-11

**Authors:** Jonas J. Lembrechts, Ann Milbau, Ivan Nijs

**Affiliations:** 1 Centre of Excellence of Plant and Vegetation Ecology, Department of Biology, University of Antwerp, Universiteitsplein 1, 2610 Wilrijk, Belgium; 2 Climate Impacts Research Centre, Department of Ecology and Environmental Science, Umeå University, SE-981 07 Abisko, Sweden; 3 Research Institute for Nature and Forest INBO, Department of Biodiversity and Natural Environment, Kliniekstraat 25, 1070 Brussels, Belgium

**Keywords:** Alien plant invasion, cold climates, disturbance, gap invasion, gradients, mountains, plant–plant interactions, stress gradient hypothesis

## Abstract

Recent experimental observations show that gap colonization in small-stature (e.g. grassland and dwarf shrubs) vegetation strongly depends on the abiotic conditions within them. At the same time, within-gap variation in biotic interactions such as competition and facilitation, caused by distance to the gap edge, would affect colonizer performance, but a theoretical framework to explore such patterns is missing. Here, we model how competition, facilitation and environmental conditions together determine the small-scale patterns of gap colonization along a cold gradient in mountains, by simulating colonizer survival in gaps of various sizes. Our model adds another dimension to the known effects of biotic interactions along a stress gradient by focussing on the trade-off between competition and facilitation in the within-gap environment. We show that this trade-off defines a peak in colonizer survival at a specific distance from the gap edge, which progressively shifts closer to the edge as the environment gets colder, ultimately leaving a large fraction of gaps unsuitable for colonization in facilitation-dominated systems. This is reinforced when vegetation size and temperature amelioration are manipulated simultaneously with temperature in order to simulate an elevational gradient more realistically. Interestingly, all other conditions being equal, the magnitude of the realized survival peak was always lower in large than in small gaps, making large gaps harder to colonize. The model is relevant to predict effects of non-native plant invasions and climate warming on colonization processes in mountains.

## Abstract (precis)

Environmental conditions within a disturbance event are often regarded as uniform. There is however large variation in conditions for plant colonisers within one disturbance event, drastically affecting coloniser survival on a scale of centimeters. We provide a model that combines the effect of the surrounding vegetation (negative through competition and positive through facilitation) with environmental conditions along a mountain gradient. Colonisers will be forced to grow closer to the gap edge when environmental conditions (e.g. freezing temperatures) get worse. The model helps predict the distribution of plant invaders and the effect of climate warming on colonisation in mountains.

## Introduction

Vegetation gaps originate from small-scale disturbances resulting in competitor-free space (Bullock 2000). They are created by a wide variety of processes, of both natural and anthropogenic origin (Chambers 1995; Bullock 2000; Kohler *et al*. 2006). Environmental conditions within gaps differ from those in the surrounding vegetation, favouring opportunistic species (Poulson and Platt 1989; Thompson *et al*. 1996; Liu and Han 2007) but disfavouring others (Thompson *et al*. 1996; Bullock 2000). In many cases, gaps, therefore, modify the realized species composition of a community (Bullock 2000; Schnitzer and Carson 2001; Vandvik 2004). This makes them important drivers of vegetation dynamics, a key process in the area of species movement under global climate change. Understanding how easily species will be able to colonize new environments requires insight in within-gap dynamics.

Worldwide, mountains undergo rapid warming, stirring debate on their susceptibility to being colonized by lowland species. Yet, gap colonization in mountains is less well understood than in lowlands because temperature gradients associated with elevation add complexity. Vegetation gaps in such dynamic systems as mountains are common and can have several causes, including animals (e.g. livestock), natural disasters (e.g. erosion, avalanches and mud slides), vegetation die-off or anthropogenic disturbances (e.g. construction works and path creation) (Chambers 1995; Körner 2003).

The survival of gap colonizers in mountains can be linked to the stress gradient hypothesis (Bertness and Callaway 1994; Maestre *et al*. 2009; Cichini *et al*. 2011). This hypothesis states that with increasing environmental harshness, facilitation gains importance over competition (Carlsson and Callaghan 1991; Callaway and Walker 1997; Callaway *et al*. 2002; Badano *et al*. 2007; Brooker *et al*. 2008; Maestre *et al*. 2009; He *et al*. 2013) because the vegetation ameliorates conditions that would otherwise limit plant growth and survival (Carlsson and Callaghan 1991; Bertness and Callaway 1994; Milbau *et al*. 2007; Wright *et al*. 2014). In particular, the plant canopy lowers wind speed, delays snowmelt and reduces net longwave radiation loss at night and in winter, overall improving minimum temperatures close to the surface (Carlsson and Callaghan 1991; Cavieres *et al*. 2007; Eränen and Kozlov 2007; Zvereva and Kozlov 2007; Abd Latif and Blackburn 2010; Cutler 2011). With regard to gap colonization, one may thus expect the surrounding vegetation to protect colonizers at the more stressful end of the gradient, so at higher elevation, whereas competition, on the other hand, would reduce colonizer survival in lowlands where abiotic stress is less severe. It needs to be noted, though, that recent data suggest that competition may remain important also at colder ends of temperature gradients (Olofsson *et al*. 1999; Forbis 2003; Eränen and Kozlov 2007; Klanderud 2010; Dvorský *et al*. 2013; Milbau *et al*. 2013).

Most research on the stress gradient hypothesis has focussed on the presence or absence of interacting neighbours, while the role of distance to neighbours has thus far been examined less often (but see Milbau *et al*. 2007; and zone-of-influence models, e.g. Jia *et al*. 2011). Yet both negative and positive interactions intensify exponentially when the distance of a colonizer to the resident vegetation diminishes (Casper *et al*. 2003; Kulmatiski and Beard 2013). The increase in competition, for example, is caused by an increasing probability of both above- and belowground space occupation and resource use by the vegetation, such as nutrient use and shading (Poulson and Platt 1989; Casper *et al*. 2003; Hu and Zhu 2008). Therefore, competition is reduced in gap centres compared with edges (Aguilera and Lauenroth 1993; Bullock 2000; Jutila and Grace 2002; Liu and Han 2007; Liu *et al*. 2008; Montgomery *et al*. 2010). In stressful environments, the presence of facilitation close to the gap edge might be essential to allow the survival of a gap colonizer. Recent experimental research in both forests and small-stature vegetation suggests that gap colonizers are indeed limited to gap edges in harsh surroundings (Heinemann and Kitzberger 2006; Cichini *et al*. 2011; Fibich *et al*. 2013; Bílek *et al*. 2014). This within-gap variation in survival conditions depending on the abiotic environment is currently largely unaccounted for in the many studies on the effects of gap size on colonization processes in forests and grasslands (e.g. Aguilera and Lauenroth 1993; Gálhidy *et al*. 2006; Liu *et al*. 2008; He *et al*. 2012).

Plants thus face high levels of competition and facilitation in gap edges, and low levels of both in gap centres. The spatial preferences of colonizers within gaps will hence depend on the relative importance of these two processes under the prevailing level of environmental harshness (e.g. cold temperatures), as well as on gap size and height and density of the surrounding vegetation, the latter of which will also depend on the environmental harshness, with smaller plant canopy heights in alpine than in lower elevation vegetation (Körner 2003 and citations therein). Understanding how these factors combine is key to accurately estimate the fate of gap colonizers in a changing environment.

In this study, we model the spatial patterns of colonizer survival inside gaps in small-stature vegetation (e.g. grassland, herbaceous vegetation or dwarf shrubs) to define the influence of the above-mentioned factors. The model is then used to predict changes in the location and magnitude of optimal survival within gaps along an elevation gradient characterized by decreasing air temperature and coinciding decreases in biotic effect size of the surrounding vegetation (smaller plants) and increases in facilitative temperature amelioration (Wright *et al*. 2015). We expect optimal survival locations to shift from the gap centre to the edge with increasing elevation, because at high elevations, the amelioration of temperature and wind stress close to the vegetation favours survival more than competition impairs it (Callaway and Walker 1997; Callaway *et al*. 2002). At the same time, we expect that the declining effect size of the surrounding vegetation towards greater elevation will diminish the fraction of the gap surface suitable for colonization.

## Methods

The survival (*S*) of gap colonizers within circular gaps in grassland or dwarf shrub vegetation under cold environmental conditions was expressed as the intrinsic survival at the prevailing minimum environmental temperature (*S*_E_), multiplied by the influences of competition (*C*) and facilitation (*F*):

(1) $$S=S_{E}CF\;$$

**Figure 1.**
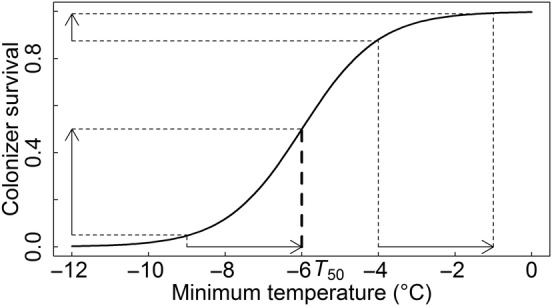
Intrinsic colonizer survival (*S*) as a function of minimum environmental temperature (*T*) for a species with a 50 % survival at a temperature of *T*_50_ = −6 °C. The effect of a local temperature amelioration of 3 °C through facilitation (Δ*T*_f_) on colonizer survival is shown for a minimum environmental temperature of −9 and −4 °C.

The intrinsic survival *S*_E_ at a minimum environmental temperature *T* itself was modelled with a logistic function based on the minimum temperature value at which survival is 50 % (*T*_50_, Fig. 1) (Larcher and Bauer 1981; Körner 2003).

(2) $$S_{E}(T)=\frac{1}{1+{\text{e}}^{-(T-T_{50})}}$$

This function incorporates some of the known and tested responses of plants to low temperatures relevant to this study: a positive exponential response with increasing temperatures at extreme temperatures and a positive linear response at moderately extreme temperatures, with maximum survival approached asymptotically in mild environments (Jame *et al*. 1999; Yan and Hunt 1999). We set *T*_50_ to −6 °C for a hypothetical species, based on the average freezing tolerance in dehardened alpine plants, experimentally obtained as the temperature at which 50 % of samples were damaged (Larcher and Bauer 1981; Körner 2003).

**Figure 2.**
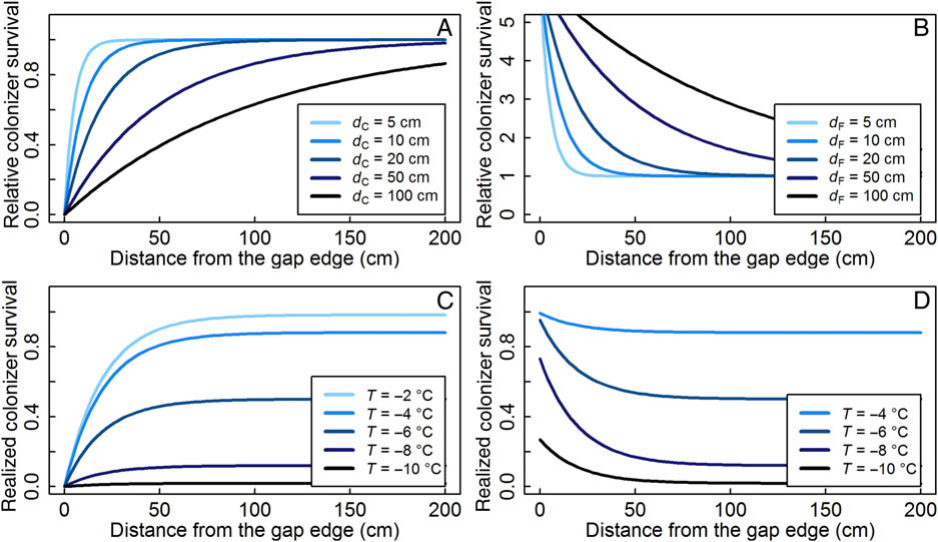
Relative (top) and realized (bottom) colonizer survival as a function of distance (*d*) to the gap edge under competition with (left) or facilitation from (right) the vegetation. (A) Relative survival under competition for varying effect sizes of the surrounding vegetation (*d*_C_). (B) Relative survival under facilitation at a minimum environmental temperature *T* of −8 °C, for varying effect sizes of facilitation (*d*_F_) and with a temperature amelioration through facilitation Δ*T*_f_ = 3 °C. (C) Realized colonizer survival under competition for varying *T*, with *d*_C_ = 20 cm. (D) Realized colonizer survival under facilitation at varying *T* with *d*_F_ = 20 cm and Δ*T*_f_ = 3 °C. *T* = −2 °C is not shown here, because the modelled temperature would shift outside the range of the model.

The relative effect of competition in Eq. (1) was modelled as an exponential function of the distance to the gap edge (*d*, in cm), based on the exponential decline in the probability of resource uptake by a plant with increasing distance from its stem base (Casper *et al*. 2003; Kulmatiski and Beard 2013). This relative competition effect equalled zero under maximal competition at a distance of 0 cm, and gradually increased (with a theoretical asymptotical maximum of 1) with increasing distance from the gap edge (Fig. 2A):

(3) $$C(d)=1-{\text{e}}^{-(d/d_{C})}$$

*C* is the relative colonizer survival under competition as a function of *d*, and *d*_C_ defines the distance from the gap edge at which this survival is reduced to 0.5. This effect size *d*_C_ defines the zone of influence of the vegetation, which correlates with its height (Zhang *et al*. 2013) and hence allows to incorporate the effect of reduced competition at high elevations indirectly through the on average smaller size of the vegetation in cold environments (Fig. 2A, Dvorský *et al*. 2013). Despite differences in competition for resources above- and belowground (Zhang *et al*. 2013), only one competitive term was used. By expressing competition as a reduction in colonizer survival, all competitive effects were combined in this one factor. Throughout the article, a *d*_C_ of 20 cm is used by default to simulate small-stature grassland or dwarf shrub vegetation. Figure 2C shows the realized survival of gap colonizers under competition on varying distances from the gap edge, in a gap with a *d*_C_ of 20 cm for a range of temperatures.

Similar to competition (Eq. 3), the influence of facilitation on survival at a certain minimum environmental temperature was modelled to decrease exponentially with increasing distance from the gap edge (Fig. 2B), dependent on the facilitative effect size of the vegetation (*d*_F_), as facilitation is as much related to the size and density of the surrounding vegetation as is competition (Eränen and Kozlov 2007). Facilitative vegetation is known to increase temperature minima and protect against freezing in cold environments (Chapin *et al*. 1979; Cavieres *et al*. 2007; Cutler 2011):

(4) $$F(d)=1+\left[\frac{S_{E}(T+\Delta T_{f})}{S_{E}(T)}\;-1\right]{\text{e}}^{-(d/d_{F})}$$

*F* and *d*_F_ were defined analogous to *C* and *d*_C_ in Eq. (3). The parameter Δ*T*_f_ represents the increase in temperature due to the cover effect of the vegetation surrounding the gap and Eq. (2) was used to calculate the intrinsic plant survival (*S*_E_). By default, a Δ*T*_f_ of 3 °C was used, a reasonable yet conservative approximation for the increase of minimum temperature through facilitation in cold environments (Cutler 2011). Later, we varied Δ*T*_f_ with elevation, implementing the known increased temperature amelioration as a function of elevation (Wright *et al*. 2015). Figure 2D shows the facilitation effect on the realized survival of a gap colonizer as a function of the distance to the gap edge for a range of environmental temperatures. The same facilitative temperature amelioration of 3 °C had a larger relative effect (*F*) in colder environments, but its realized effect decreased again in the most extreme environments, due to the lower values of *S* (Fig. 2D, Brooker *et al*. 2008).

**Figure 3.**
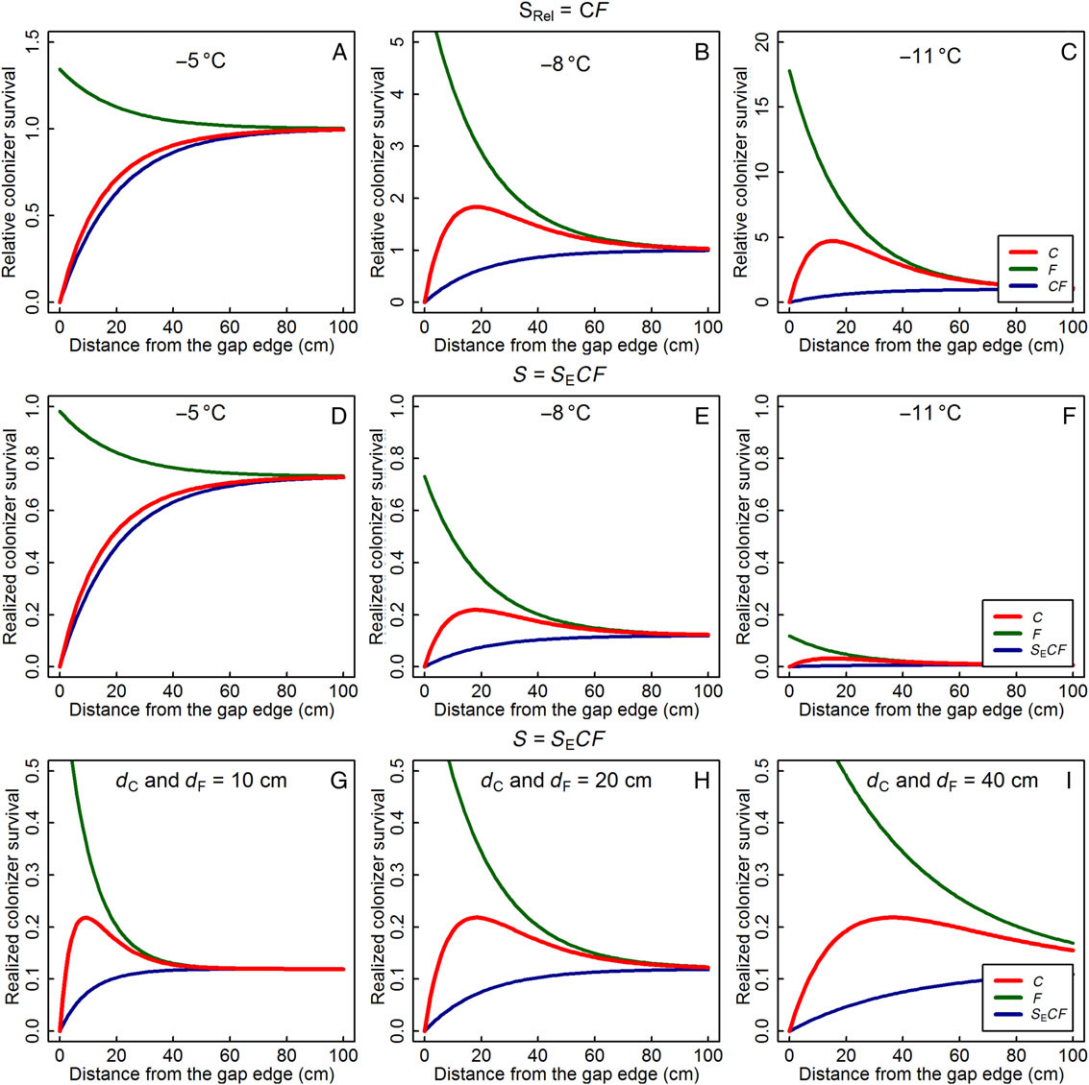
Top and middle row: relative (*S*_Rel_, A–C) and realized (*S*, D–F) colonizer survival (red) with changing distance to the gap edge (cm) for *T* = −5 °C (A and D), −8 °C (B and E) or −11 °C (C and F). Δ*T*_f_ = 3 °C, *d*_C_ and *d*_F_ = 20 cm. Bottom row: realized colonizer survival with *d*_C_ and *d*_F_ = 10 cm (G), 20 cm (H) or 40 cm (I). *T* = −8 °C, Δ*T*_f_ = 3 °C. The underlying components of competition (Function *C*) and facilitation (Function *F*) are shown, respectively, in blue and green, their product in red. See Fig. 2 for other symbols, and text for used functions. The *y*-axis is variable on the top row, set to 1 on the middle row and set to 0.5 on the bottom row.

Subsequently, the previous equations were combined to model the relative (*S*_Rel_) and realized (*S*) survival at a distance *d* from one vegetation edge: *S*_Rel_ = *CF*, *S* = *S*_E_*CF* (Fig. 3). This relative survival (*S*_Rel_) under competition and facilitation was thus multiplied with the intrinsic survival (*S*_E_) to calculate the realized survival (*S*).

In the following simulations within circular gaps, the effects of competition and facilitation were integrated for each location in the gap by taking the average of all calculated colonizer survival values for all possible distances to the gap edge in circular gaps of varying sizes. We used gaps with sizes ranging from 5 cm to 1 m to include both small-scale natural gaps (as caused by animals for example) and larger scale disturbances as caused by humans or natural disasters. Although many of these disturbances will in reality have irregular shapes or will even be linear (like trails), we chose for circular gaps for simplicity and general applicability of the theoretical insights. As the biotic effects will fade out after a certain distance, it is not necessary to model larger gaps, as colonizer survival will stay constant after a certain distance (*S* = *S*_E_).

We first simulated a gap without freezing (0 °C) and thus without facilitative effect, only taking into account competition. Next, survival was modelled for harsher conditions at −8 °C, with both competition and facilitation, and for different gap sizes to show the effect of gap sizes on the survival of gap colonizers.

All parameters in Eqs. (1–4) were subsequently varied separately at a fixed gap size to unravel their individual effects and understand their roles in shaping survival patterns. We separately varied the environmental temperature (*T*), the colonizer characteristics (*T*_50_) and the characteristics of the surrounding vegetation (*d*_C_, *d*_F_ and Δ*T*_f_).

Finally, we calculated gap colonization along a realistic temperature gradient in mountains by decreasing the minimum environmental temperature, the effect size of the vegetation with increasing elevation (representing the decreasing vegetation size in colder conditions; Körner 2003) and increasing the facilitative temperature amelioration (Wright *et al*. 2015). More precisely, we modelled an elevational gradient of ∼2000 m with a minimum temperature shift from *T* = −4 to −12 °C (Minder *et al*. 2010) from the lowest to the highest elevation, a corresponding decrease in effect sizes of both facilitation and competition from *d*_C_ = *d*_f_ = 50 to 5 cm and a change in facilitative temperature amelioration from Δ*T*_f_ = 1 to 5 °C.

All simulations were run in R (R Development Core Team 2013).

## Results

The graphs of the combined relative (*S*_Rel_) and realized (*S*) survival with increasing distance to one gap edge (Fig. 3A–F) show the trade-off between competition and facilitation with distance and the changes in realized survival with decreasing minimum temperatures. With more severe frost, maximal survival occurred closer to the edge due to the higher relative importance of facilitation (Fig. 3A–C). At the same time, the absolute values of the maxima decreased as lower temperatures reduced intrinsic survival (Fig. 3D–F), first limiting it to locations close to the edge (Fig. 3E) and ultimately reducing survival to virtually zero everywhere in the gap at *T* = −11 °C (Fig. 3F). A greater effect size of the vegetation shifted the optimum away from the edge, and enhanced survival across a greater range (Fig. 3G–I).

**Figure 4.**
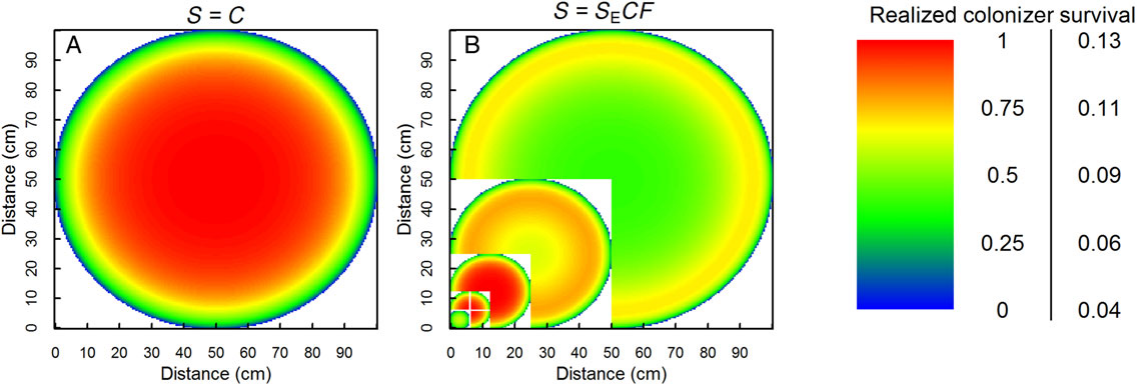
Realized colonizer survival as a function of distance to the gap edge (cm) with competition at positive temperatures within a gap of 100 cm diameter (A), and with facilitation and competition at *T* = −8 °C in gaps of different sizes (B). Note the different colour scales for (A) (left) and (B) (right). *d*_C_ = *d*_F_ = 20 cm, Δ*T*_f_ = 3 °C. See Figs 2 and 3 for other symbols.

When modelling gap survival in an environment without sub-zero temperatures and hence an intrinsic survival of 1, the realized colonizer survival increased asymptotically towards the gap centre (Fig. 4A). The inclusion of facilitation and lower intrinsic survival in sub-zero temperatures, on the other hand, created variable patterns in which the location of optimal survival depended on the gap size (shown for −8 °C in Fig. 4B). In small gaps, realized survival was still maximal in gap centres, similar to Fig. 4A, but beyond a certain gap size (*d* > 25 cm at *T* = −8 °C), realized colonizer survival unexpectedly decreased in the entire gap. This decrease was faster in gap centres than edges, where survival remained higher due to the positive effect of facilitation. The highest survival rates, however, occurred in gaps of intermediate size (*d* around 25 cm in Fig. 4B).

**Figure 5.**
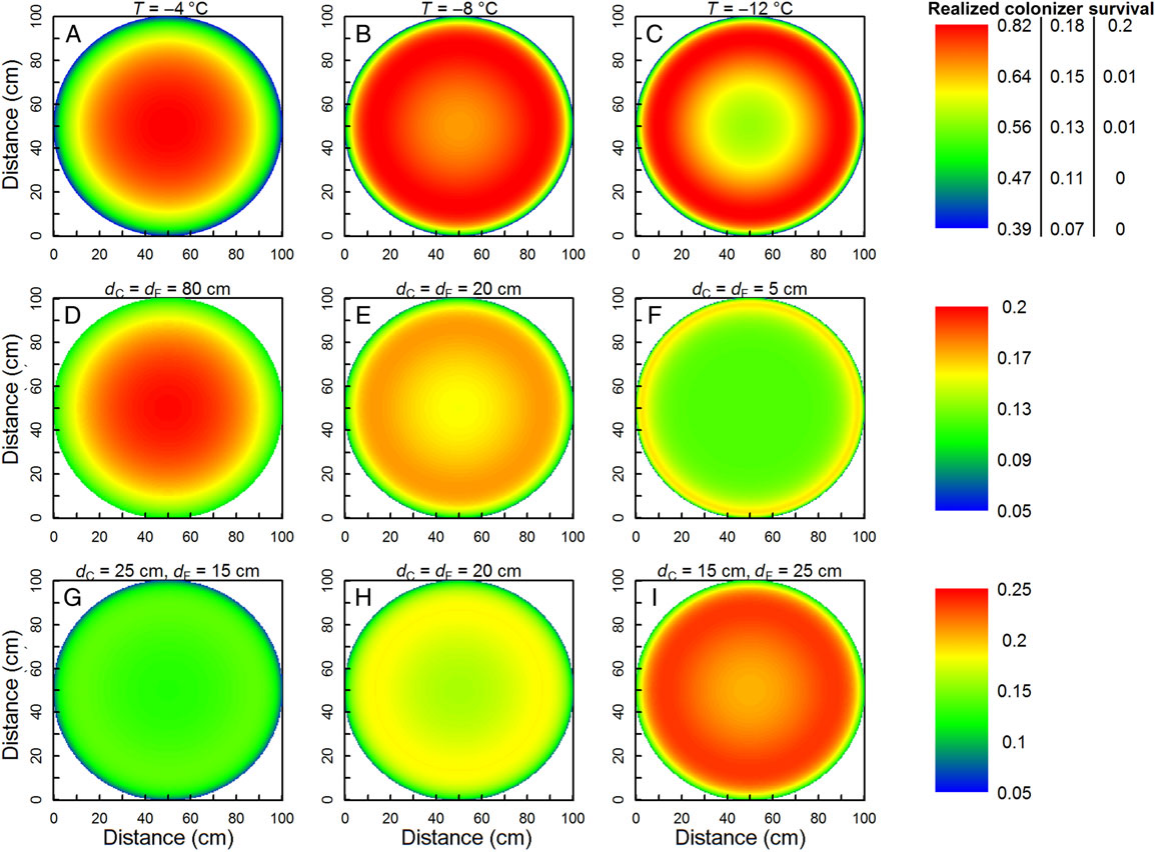
Realized colonizer survival as a function of distance to the gap edge for gaps of 100 cm diameter. Top row: *T* = −4, −8 or −12 °C with *d*_C_ = *d*_F_ = 20 cm. Middle row: *d*_C_ and *d*_F_ of 80, 20 and 5 cm at *T* = −8 °C. Bottom row: *d*_C_ = 25 cm and *d*_F_ = 15 cm, *d*_C_ = *d*_F_ = 20 cm, *d*_C_ = 15 cm and *d*_F_ = 25 cm at *T* = −8 °C. Δ*T*_f_ was always 3 °C. See Fig. 2 for symbols. Note the different colour scales within the first row and between the rows.

After showing the effect of competition and facilitation as a function of distance to one gap edge (Fig. 3) and the effects of varying gap sizes (Fig. 4B), we varied the effects of other parameters in gaps of a constant diameter of *d* = 100 cm. The exact location and value of maximal colonizer survival within gaps depended on the minimum environmental temperature (*T*), the colonizer's sensitivity to low temperatures (*T*_50_) and the characteristics of the vegetation (*d*_C_, *d*_F_ and Δ*T*_f_). With declining *T*, colonizer survival in gaps of equal size dropped, but faster in gap centres than edges, which is similar to the decline observed with increasing gap sizes (Fig. 5A–C, note the different scaling). Colonizers in cold environments were thus increasingly restricted to gap edges (Fig. 5C). Conversely, at less extreme temperatures, the importance of temperature facilitation declined while competition remained, resulting in better survival in gap centres. Moreover, the gap surface available for colonization was significantly larger at high compared with low temperatures (Fig. 5A–C). Changing the colonizer's *T*_50_ resulted in species-specific shifts of the whole pattern along the environmental gradient, yielding exactly the same outcomes but at different temperatures. Species with a lower *T*_50_ performed relatively better at the same minimum temperature (as shown for *T* = −8 °C in **Supporting Information—Fig. S1**). Adding an extra factor to Eq. (2) by replacing (*T* − *T*_50_) by a(*T* − *T*_50_) and varying *a* changed the steepness of the species' temperature reaction curve **[see** **Supporting Information—Fig. S1****]**. Different species then had different survival optima within gaps along the gradient.

In our model, the size and density of the surrounding vegetation were represented by the effect sizes of competition and facilitation (*d*_C_ and *d*_F_) and the temperature increase through facilitation (Δ*T*_f_). In vegetation with larger *d*_C_ and *d*_F_, maximal survival occurred further away from the gap edge, as already observed in Fig. 3 (Fig. 5D–F, at *T* = −8 °C), and a large effect size made large parts of the edges less suitable for colonization because competition dominated over facilitation (Fig. 5D). Small values of *d*_C_ and *d*_F_, on the other hand, resulted in lower survival in gap centres and relatively higher survival in the edges (Fig. 5F). Dissimilar values for *d*_C_ and *d*_F_ surprisingly affected the overall survival more than the location of the optima (Fig. 5G–I, note a different scaling compared with Fig. 5D–F). Survival was especially low in vegetation with large competitive and small facilitative influences. Changing the size of the facilitative temperature increase (Δ*T*_f_) altered the realized survival, but not the location of peak survival nor the overall response pattern **[see** **Supporting Information—Fig. S2****]**. Higher values of Δ*T*_f_ increased the peak survival, while lower values had the opposite effect.

**Figure 6.**
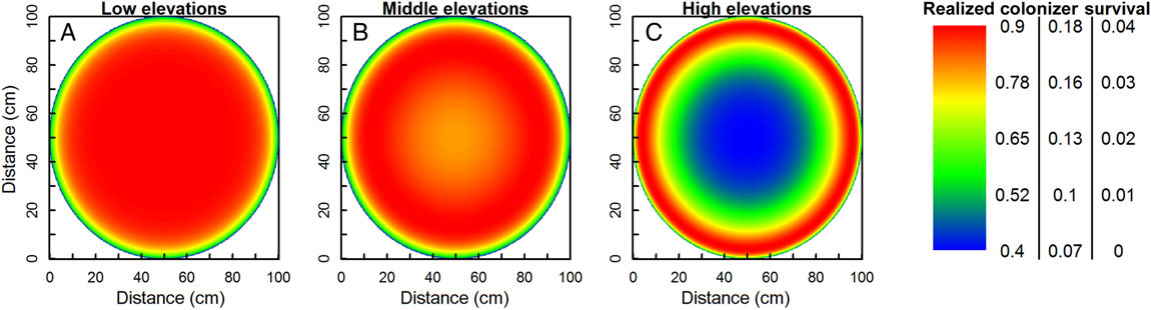
Realized colonizer survival along a theoretical elevational gradient as a function of distance to the gap edge for gaps of 100 cm diameter, for low (*T* = −4 °C, *d*_C_ = *d*_F_ = 50 cm, Δ*T*_f_ = 1 °C), middle (*T* = −8 °C, *d*_C_ = *d*_F_ = 20 cm, Δ*T*_f_ = 3 °C) and high (*T* = −12 °C, *d*_C_ = *d*_F_ = 5 cm, Δ*T*_f_ = 5 °C) elevations. See Fig. 2 for symbols. Note the different colour scales.

**Figure 7.**
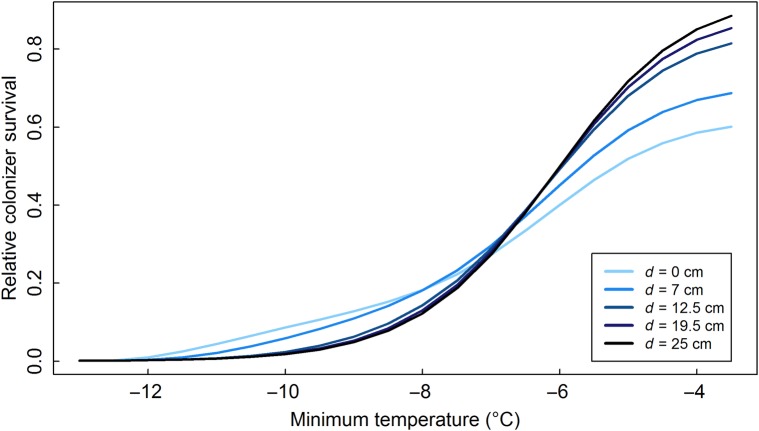
Realized colonizer survival at different distances from the gap edge (*d* in cm, from the gap edge (*d* = 0 cm, light blue line) till 25 cm from the gap edge (*d* = 25 cm, dark line)) for gaps of 100 cm diameter, along a theoretical elevational gradient (*x*-axis). Temperatures along this gradient range from *T* = −13 °C to −3 °C. *d*_*C*_ = *d*_*F*_ covary along this gradient, ranging from 50 to 5 cm, *ΔT*_*f*_ from 5 °C to 1 °C.

As the effect sizes of competition and facilitation and the size of the facilitative temperature amelioration covary with temperature along a real mountain gradient, we modelled their interaction (Fig. 6). More extreme minimum environmental temperatures together with smaller vegetation but on average higher facilitative temperature amelioration increased the eccentricity of maximal survival on higher elevations even more than when all of them were considered separately. Colonizer survival was reduced and limited to the edges, and a large fraction of the gap surface became unsuitable for colonizers at high elevations. At low elevations, on the other hand, the pattern was opposite, with most parts of the gap surface at a certain distance from the edge available for colonization. These interactions resulted in an overall decrease in colonizer survival with decreasing minimum temperatures, albeit at a slower pace in gap edges than in gap centres, shifting the location of the optimum from the gap centre to the gap edge before ultimately reducing colonizer survival to zero in the whole gap (Fig. 7).

## Discussion

### Within-gap survival optimum

The stress gradient hypothesis predicts a shift from competition to facilitation-dominated systems along a stress gradient (Bertness and Callaway 1994). Our model adds another dimension by focussing on the within-gap environment, showing that the interaction between competition and facilitation defines a peak in colonizer survival at a certain distance from the gap edge. This optimal distance depends on the minimum environmental temperature and characteristics of the surrounding vegetation, the importance of which has also been shown in experimental studies (e.g. Cichini *et al*. 2011). Our model predicts that colonizer survival decreases on all locations in the gap with increasing environmental harshness, but at a slower pace in gap edges (Fig. 7). The location of optimal survival hence shifts from gap centres to gap edges in cold environments, leaving large parts of gaps unavailable for colonizers, while plants will grow in a more aggregated way (see e.g. Kikvidze *et al*. 2005). This eccentricity is enhanced when we accounted additionally for smaller vegetation with smaller effect sizes of competition and facilitation in colder temperatures, but a relatively higher temperature amelioration (Wright *et al*. 2015). These results trace back to our assumption that the relative importance of facilitation increases at lower temperatures (Brooker and Callaghan 1998; Wright *et al*. 2015), while the relative intensity of competition stays the same. Indeed, competition depends on the ability of the vegetation to take away resources, which is determined more by plant size than by temperature (Brooker and Callaghan 1998; Dormann and Brooker 2002; Forbis 2003). As temperatures continue to drop, the interaction between critically low intrinsic survival and much reduced vegetation size can also explain why experimental studies ultimately observe reduced facilitation under extreme environmental harshness (Brooker *et al*. 2008). Here, facilitation will no longer overcome environmental limitations.

### Effects of gap size and species-specific characteristics

As observed experimentally (Liu *et al*. 2008; Tozer *et al*. 2008), the location and magnitude of the optimal survival predicted by the model depended on gap size, following a hump-shaped pattern with increasing gap size. In small gaps (and in warm environments), gap size and colonizer survival were positively correlated, with highest survival in gap centres. Such a preference for gap centres has also been shown before (Poulson and Platt 1989; Jutila and Grace 2002; Heinemann and Kitzberger 2006; Fibich *et al*. 2013). For larger gaps, however, we observed an increasingly eccentric location of survival optima.

Species at the edge of their temperature niche (in this case, approaching environmental minimum temperatures of −12 °C, Figs 1 and 7) are particularly hampered in gap centres. This pattern is supported by observations in cold-climate ecosystems in general, where only small gaps and gap edges close to the established vegetation stay available for colonizers (Carlsson and Callaghan 1991; Eränen and Kozlov 2007; Cichini *et al*. 2011; Milbau *et al*. 2013). Interestingly, in our model also, the magnitude of the realized survival optimum was lower in larger gaps, regardless of temperature, making them harder to colonize in facilitation-dominated systems. This is linked to the fact that colonizers of small gaps not only experience the facilitation effect from the closest gap edge, but from the whole gap. In large gaps, on the other hand, only the closest gap edge adds to the facilitative effect. For these reasons, recolonization after large-scale disturbance of natural or anthropogenic origin would be restricted in cold environments (Eränen and Kozlov 2007), slowing down the recovery of the system. As anthropogenic disturbances (e.g. road construction) will often be even larger in size than shown here, our model predicts colonization of these disturbances in grassland or dwarf shrub vegetation in cold environments to be severely hampered.

Varying the species-specific parameters of the model demonstrated that species with a different temperature response had survival optima at different locations within gaps. This finding links to niche differentiation and the gap partitioning hypothesis, stating—for forest gaps—that different colonizers will prefer different gap parts (Busing and White 1997; Kern *et al*. 2013). Species less adapted to the environmental conditions can indeed still outcompete better adapted species at the edge of a gap, owing to facilitation (Busing and White 1997; Ritter *et al*. 2005; Prévost and Raymond 2012). It is important to notice that the model only focusses on the first stages of establishment, as larger plants in later successional stages can facilitate their own survival as much as the surrounding vegetation does (Körner 2003).

### Other environmental stress gradients

While we focussed on cold gradients in mountains, the model can likewise be applied to latitudinal cold gradients, where plant size and temperature also decrease simultaneously towards high-latitude systems. Although the model only incorporated a temperature gradient, it does integrate the indirect effects of cold on other environmental conditions. Nutrient levels are, for example, often limiting at high elevations (Körner 2003), but these add to the reduced survival potential (*S*_E_) in cold environments, in line with the modelled patterns. The same holds true for shading, which will play a less important role at high elevations and is included in the declining effect size of competition exerted by the lower vegetation at high elevation.

The model only considers an environmental severity gradient for one non-resource condition (minimum temperature in mountains) and its direct and indirect effects on competition and facilitation. It could be recalculated, however, for other types of facilitation on other stress gradients, such as attracting nutrients or cooling by providing shade, which are likely to be important in respectively nutrient poor and hot environments, also occurring in mountains (Callaway and Walker 1997; Holmgren and Scheffer 2010; Madrigal-González *et al*. 2013; Michalet *et al*. 2014). For facilitation that decreases the maximum temperature such as shading, this can be implemented easily by defining a decreasing survival-temperature curve rather than the increasing one shown in Fig. 1. He *et al*. (2013) observed in their meta-analysis a consistent trend towards growing closer to the vegetation in the case of stress due to resource limitations, but stated that this was caused by a reduction in competition more than by an increase in facilitation. We conjecture that the same patterns will occur along all types of stress gradients: the harsher the conditions, the more colonizers will be limited to gap edges.

### Assumptions and validation

We addressed the process of gap colonization in mountains with mathematical modelling, neglecting the colonizers' demography. This avoids the complexity and excessive run time of simulations associated with identifying separate individuals, but more important is that introducing demographic parameters is unlikely to alter the outcome. For example, few or no colonizers will establish on locations in the gap where conditions do not allow it, even when seed supply is abundant. Likewise, growth and mortality will correlate with the same environmental and biotic variables that determine the modelled colonizer survival. Our underlying mathematical approach is based on the suitability of gaps for colonizer survival and the variation in conditions within those gaps by focussing on the small-scale zonation and environmental variation within them. By applying macro-ecological principles on a micro-scale, we highlight the importance of strong environmental gradients on ecological processes on a micro-scale in gaps, as also observed in other systems (Bennie *et al*. 2008). Other assumptions, chosen equations and values were justified in the Methods.

The model could be validated by measuring gap colonizer survival along an elevation gradient together with some basic abiotic variables and gap characteristics. Minimum temperatures for plant survival are often reported (e.g. Körner 2003 and references therein), and these temperatures are easy to record in the field with temperature loggers, even on a small scale. The Δ*T*_f_ parameter then results from the difference in temperature between edge and centre, and the effect size of facilitation will be visible as the distance from the gap edge where the increase in minimum temperature is still at 50 %. The effect size of competition can be derived from measurements of light reduction in the gap edge. As in reality gaps will not be circular and effect sizes will vary depending on the orientation and inclination of the sun, real-life patterns will be less straightforward than shown here. However, as our model estimates survival for every gap position separately by calling the function every time again, this real-life variation can be implemented when needed.

### Applications

The conclusions of the model are relevant for two major global change challenges in mountains: non-native plant invasions and climate change. Non-native plant diversity in mountains is currently strongly correlated with the competitive release provided by disturbance, such as in roadsides that can be interpreted as large-scale linear gaps (Seipel *et al*. 2012; Lembrechts *et al*. 2014). Based on our model, however, the harsh climate at high elevations limits non-native plant survival in gaps and open spaces, while facilitation becomes the key driver of their success (Cavieres *et al*. 2005; Badano *et al*. 2007; Quiroz *et al*. 2011). A large part of those disturbed areas hence becomes unavailable for non-native species, as they stay limited to small gaps (Milbau *et al*. 2013) and locations close to the established vegetation. This theory contributes to the explanation of why plant invasion along mountain roads slows down with elevation (Alexander *et al*. 2011; Seipel *et al*. 2012). It may also explain why at high elevations a larger fraction of non-native species from roadsides can be found in the undisturbed vegetation (Lembrechts *et al*. 2014). Based on our modelled patterns, we thus warn that, contrary to commonly assumed, non-native species might become less connected to large, mostly anthropogenic, disturbances at high elevations, as their survival chances will be higher in small, natural gaps in the natural vegetation. This shift from a limited number of locations of high disturbance to the vast area of less disturbed nature in mountains might make invasion management more challenging.

The spatial temperature gradient in mountains can also be construed as a temporal one. From this perspective, the strong warming in alpine environments resulting from climate change (Körner 2003) might decrease the importance of facilitation at the expense of competition (Klanderud 2010). Our results indicate that gap colonizers will then get opportunities to use a larger portion of gap surfaces, increasing the efficiency of gap regeneration and the succession rate in disturbed alpine environments in a warmer climate. Increasing anthropogenic disturbance in mountains, in combination with climate change, might as such accelerate the observed upward movement of several species (Pauli *et al*. 2007). Non-native species might also profit from this trend in a warmer climate, as it will increase their ability to use linear anthropogenic disturbances as pathways to higher elevations (Pauchard *et al*. 2009) and fill in the gaps created by disturbance processes in mountains. The greater importance of competition, on the other hand, would increase the threshold of the minimal gap size for successful colonization, although our results show that this effect will be secondary.

## Conclusions

This model provides a framework for future research on facilitation and competition in gaps created by natural or anthropogenic disturbance and helps predicting colonization processes in conditions of varying environmental harshness. With the help of a mathematical approach, it connects the research on gap regeneration with the vast literature on biotic interactions and the stress gradient hypothesis.

The focus on within-gap variation in growing conditions highlights the need for more detailed studies of small-scale climatic and biotic responses to explain and predict large-scale processes, as the inclusion of small-scale variation in this model indicates that the use and recolonization of open areas after disturbance might very well be less straightforward than often assumed. The model, and future experimental studies building on it, can help understand and predict global phenomena such as non-native plant invasion and the effects of disturbance under climate change in cold-climate mountain ecosystems.

## Sources of Funding

J.J.L. received support from the Methusalem Programme of the Flemish Government via the PLECO research group and a grant from the Research Foundation—Flanders (FWO).

## Contributions by the Authors

All authors contributed to model development and paper writing.

## Conflict of Interest Statement

None declared.

## Supporting Information

The following additional information is available in the online version of this article –

**Figure S1.** (A) Intrinsic colonizer survival (*S*) as a function of minimum environmental temperature (*T*) for a species with a range of 50 % survival temperatures: *T*_50_ = −4 °C till −8 °C. (B–D) Realized colonizer survival as a function of distance to the gap edge for gaps of 100 cm diameter. (B) *T*_50_ = −5 °C, (C) *T*_50_ = −6 °C, (D) *T*_50_ = −7 °C with *d*_C_ = *d*_F_ = 20 cm, *T* = −8 °C and Δ*T*_f_ = 3 °C. (E) Intrinsic colonizer survival (*S*) as a function of minimum environmental temperature (*T*) for a species with *T*_50_ = −6 °C and the correction factor *a* varying from 0.25 to 4. (F and G) Realized colonizer survival as a function of distance to the gap edge for gaps of 100 cm diameter with *a* = 0.5 (F), 1 (G) or 2 (H).

**Figure S2.** Realized colonizer survival as a function of distance to the gap edge for gaps of 100 cm diameter with varying Δ*T*_f_ ranging from 1 to 5 °C. *d*_C_ = *d*_F_ = 20 cm, *T* = −8 °C, *T*_50_ = −6 °C.

Additional Information
