# Supplementary material for: Trade-off between competition and facilitation defines gap colonization in mountains
Source: AoB Plants. 2015 Nov 11;7:plv128. doi: 10.1093/aobpla/plv128 (PMC4683995; doi:10.1093/aobpla/plv128)
Supplement: Additional Information [file supp_7_plv128_index.html]

Trade-off between competition and facilitation defines gap colonisation in mountains — Trade-off between competition and facilitation defines gap colonization in mountains — Additional Information 

# Trade-off between competition and facilitation defines gap colonization in mountains

## Additional Information

Additional Information

- Additional Information - Docx file
