# Supplementary material for: Trade-off between competition and facilitation defines gap colonization in mountains
Source: AoB Plants. 2015 Nov 11;7:plv128. doi: 10.1093/aobpla/plv128 (PMC4683995; doi:10.1093/aobpla/plv128)

**APPENDIX**

**Figure A.** A. Intrinsic coloniser survival (S) as a function of minimum environmental temperature (T) for a species with a range of 50% survival temperatures: T_50_ = -4 °C till -8 °C. B-D. Realised coloniser survival as a function of distance to the gap edge for gaps of 100 cm diameter. B: T_50_ = -5 °C, C: T_50_ = -6 °C, D: T_50_ = -7 °C with d_C_ = d_F_ = 20 cm, T = -8 °C and ΔT_f_ = 3 °C. E. Intrinsic coloniser survival (S) as a function of minimum environmental temperature (T) for a species with T_50_ = -6 °C and the correction factor a varying from 0.25 to 2. F-G. Realised coloniser survival as a function of distance to the gap edge for gaps of 100 cm diameter with a = 0.5 (F), 1 (G) or 2 (H).

**Figure B.** Realised coloniser survival as a function of distance to the gap edge for gaps of 100 cm diameter with varying ΔT_f_ ranging from 1 to 5 °C. d_C_ = d_F_ = 20 cm, T = -8 °C, T_50_ = -6 °C.


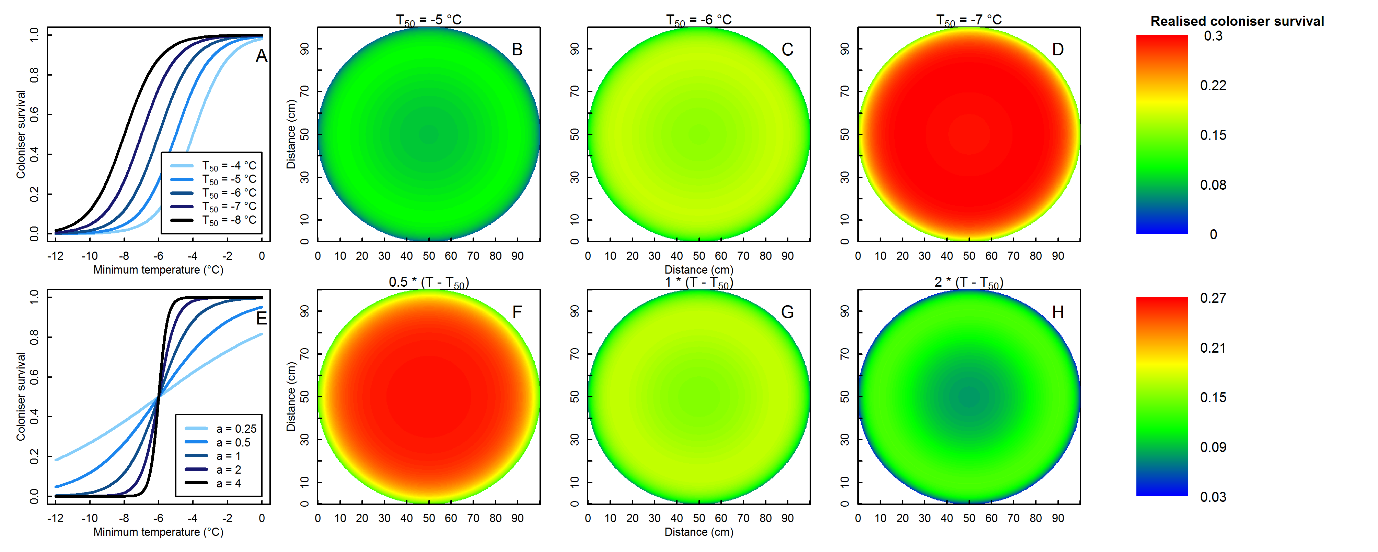


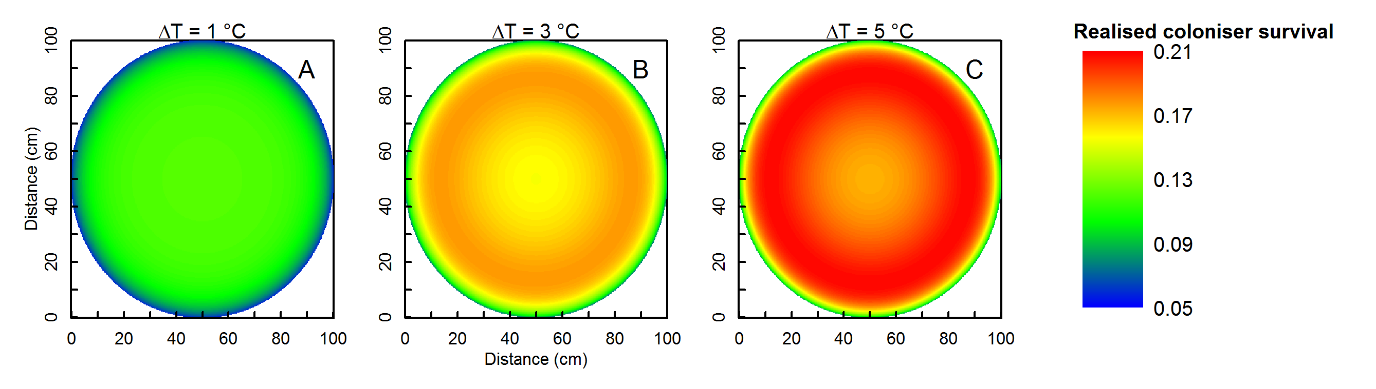

Supplement: Additional Information [file supp_plv128_plv128supp.docx]
